# Leprosy mimicking lymphoproliferative disorders: a case report and structured literature search

**DOI:** 10.1016/j.ijregi.2026.101029

**Published:** 2026-09-26

**Authors:** Laís Agostinetto, Caroline Goergen, Amanda Hillesheim Schuck, Gabriela de Oliveira Centofante, Katiane Bortolini Zenatti, Janine Alessi

**Affiliations:** 1 Internal Medicine Division, São Lucas Hospital, Pontifical Catholic University of Rio Grande do Sul, Porto Alegre, Brazil; 2 Postgraduate Program in Medicine and Health Sciences, Pontifical Catholic University of Rio Grande do Sul, Porto Alegre, Brazil; 3 School of Medicine, Pontifical Catholic University of Rio Grande do Sul, Porto Alegre, Brazil; 4 Institute of Pathology, Porto Alegre, Rio Grande do Sul, Brazil; 5 Postgraduate Program in Biomedical Gerontology, Pontifical Catholic University of Rio Grande do Sul, Porto Alegre, Brazil

**Keywords:** Borderline leprosy, Endemic disease, Multibacillary borderline leprosy, Infectious disease, Case report

## Abstract

**Objectives:**

Leprosy is a chronic infectious disease caused by *Mycobacterium leprae* that remains endemic in several regions, including Brazil. The disease predominantly affects the skin and peripheral nerves, but atypical presentations dominated by systemic symptoms such as fever and lymphadenopathy, particularly when accompanied by nonclassical cutaneous findings, are rare and may be mistaken for lymphoproliferative disorders. This study aim to raise clinical suspicion for atypical leprosy among health care providers in endemic areas, complemented by a structured literature search of comparable published cases to better characterize this diagnostic pitfall.

**Methods:**

This study was conducted in two steps. First, we reported an index case of multibacillary borderline leprosy in accordance with the CAse REport (CARE) guidelines. Second, we performed a structured literature search across MEDLINE, Embase, and the Cochrane Library to identify published case reports and case series describing leprosy presenting as, or mimicking, lymphoma or another lymphoproliferative disorder.

**Case presentation:**

We describe a 44-year-old woman from southern Brazil with a 1-year history of progressive, initially painful, generalized lymphadenopathy (inguinal, cervical, and axillary), a diffuse pruritic erythematous eruption, and systemic B symptoms (fever, night sweats, and 9 kg of unintentional weight loss). An outside skin biopsy had been read as psoriasiform dermatitis, and the patient had been treated unsuccessfully with methotrexate. One year later, skin and lymph node biopsies subsequently demonstrated acid-fast bacilli on Ziehl–Neelsen staining and histopathology consistent with Hansen's disease, establishing a diagnosis of multibacillary borderline leprosy. The patient was started on multidrug therapy (rifampicin, clofazimine, dapsone). Skin lesions progressively improved and constitutional symptoms resolved with treatment, although residual neuropathic symptoms persisted. Household contact investigation identified a sister with compatible skin lesions.

**Results:**

Of 785 records screened, 20 underwent full-text assessment; 13 case reports described a comparable diagnostic pitfall. The closest parallels involved generalized lymphadenopathy with a full constitutional syndrome misattributed to tuberculosis or lymphoma (Bagla, Mahajan, Singh, Herekar, Kiran, and especially Reis et al., a Brazilian case with an almost identical systemic symptom profile); a second group mimicked lymphoma cutaneously (Gunawan, Nawalerspanya, Rodríguez et al.). Taken together, our structured literature search identified relatively few reports combining, as our patient did, disseminated multi-chain lymphadenopathy and a full constitutional syndrome. This combination underscores both the rarity of such a comprehensive presentation and the persistent risk of diagnostic delay when leprosy is not considered in patients from endemic regions presenting with lymphadenopathy and systemic symptoms, reinforcing the educational aim of this case report and structured literature search.

**Conclusion:**

This case and accompanying structured literature search highlight leprosy as an important, underrecognized mimicker of lymphoproliferative disorders when it presents with systemic symptoms and lymphadenopathy. Increased clinical awareness, particularly in primary care and endemic settings, along with systematic contact tracing, is essential to reduce diagnostic delay and interrupt transmission.

## Highlights

• First case report paired with a systematic review of leprosy mimicking lymphoma.
• Synthesizes 13 published cases into a single recognizable diagnostic phenotype.
• Defines two mimicry patterns: nodal-constitutional and cutaneous lymphoma-like.
• Documents a triad of multi-chain adenopathy, B symptoms, and misdiagnosis.
• Provides evidence to shorten diagnostic delay in leprosy-endemic settings.

## Background

Leprosy is a chronic infectious disease caused primarily by *Mycobacterium leprae*, which remains endemic in several regions of the world and in Brazil [1]. The World Health Organization reports that in 2023, a total of 182,815 new cases were reported globally, corresponding to a new case detection rate of 22.7 per million population. This represented a 5% increase compared to 2022 (174,094 cases) [2]. The primary mode of transmission is through prolonged contact with untreated individuals with high bacillary loads, particularly via respiratory secretions [1]. The disease predominantly affects the skin and peripheral nerves, leading to varying degrees of neuropathy.

Borderline leprosy refers to the intermediate forms of leprosy in the Ridley-Jopling classification system, representing an immunologically unstable spectrum between the two polar forms (tuberculoid and lepromatous). The borderline forms include borderline tuberculoid, borderline-borderline, and borderline lepromatous leprosy [3]. The borderline forms are characterized as "immunologically unstable" within this continuum, with the ability to move toward tuberculoid or move toward lepromatous forms based on changes in cell-mediated immunity [3]. While fever and lymphadenopathy can occur in leprosy, they are rare as the sole or primary presenting features. Atypical presentations of leprosy may lead to diagnostic delays ranging from 12 to 96 months (mean of 22 months), with isolated neurological symptoms associated with the longest intervals (mean of 38-40 months) [4,5].

Despite the importance of early diagnosis and treatment, presentations of leprosy limited to systemic manifestations such as fever and lymphadenopathy, without the classical findings, are particularly difficult to recognize, as primary health care professionals often lack sufficient training to consider Hansen's disease as a differential diagnosis for isolated constitutional symptoms [1]. By detailing the diagnostic challenges encountered in this case, we aim to raise clinical suspicion for atypical leprosy among health care providers in endemic areas, complemented by a structured literature search of comparable published cases to better characterize this diagnostic pitfall.

## Methods

### Case report

This manuscript describes an index case of multibacillary borderline leprosy presenting with generalized lymphadenopathy and systemic B symptoms (fever, night sweats, and unintentional weight loss) that raised clinical suspicion for lymphoma prior to histopathological confirmation of Hansen's disease. The case was reported in accordance with the CARE (CAse REport) guidelines for the transparent and complete reporting of clinical case reports [6]. A completed CARE checklist indicating where each reporting item is addressed in the manuscript is provided in Supplementary Table 1.

### Structured literature search

To contextualize the index case within the existing body of evidence, we conducted a structured literature search to identify published case reports and case series describing leprosy presenting as, or mimicking, lymphoma or another lymphoproliferative disorder.

A comprehensive literature search was performed across three electronic databases: MEDLINE, Embase, and the Cochrane Library, from database inception to April 23, 2026, with no restrictions on language or publication date. The complete search strategies used for each database are provided in Supplementary Table 2.

Eligible publications included case reports and small case series describing patients with a confirmed diagnosis of leprosy (Hansen’s disease). Reports were considered eligible when leprosy initially presented as, was suspected to represent, or clinically mimicked lymphoma or another lymphoproliferative disorder. Narrative reviews, systematic reviews, scoping reviews, and meta-analyses were excluded, as were experimental and observational studies. Publications describing the coexistence of leprosy and lymphoma were also excluded when there was no evidence that leprosy had mimicked the neoplasm. Conference abstracts without a corresponding full case report publication were also excluded.

The identification, screening, eligibility assessment, and inclusion of reports were documented using a Preferred Reporting Items for Systematic Reviews and Meta-Analyses 2020 flow diagram to transparently depict the study selection process (Figure 1) [7].

**Figure 1:**
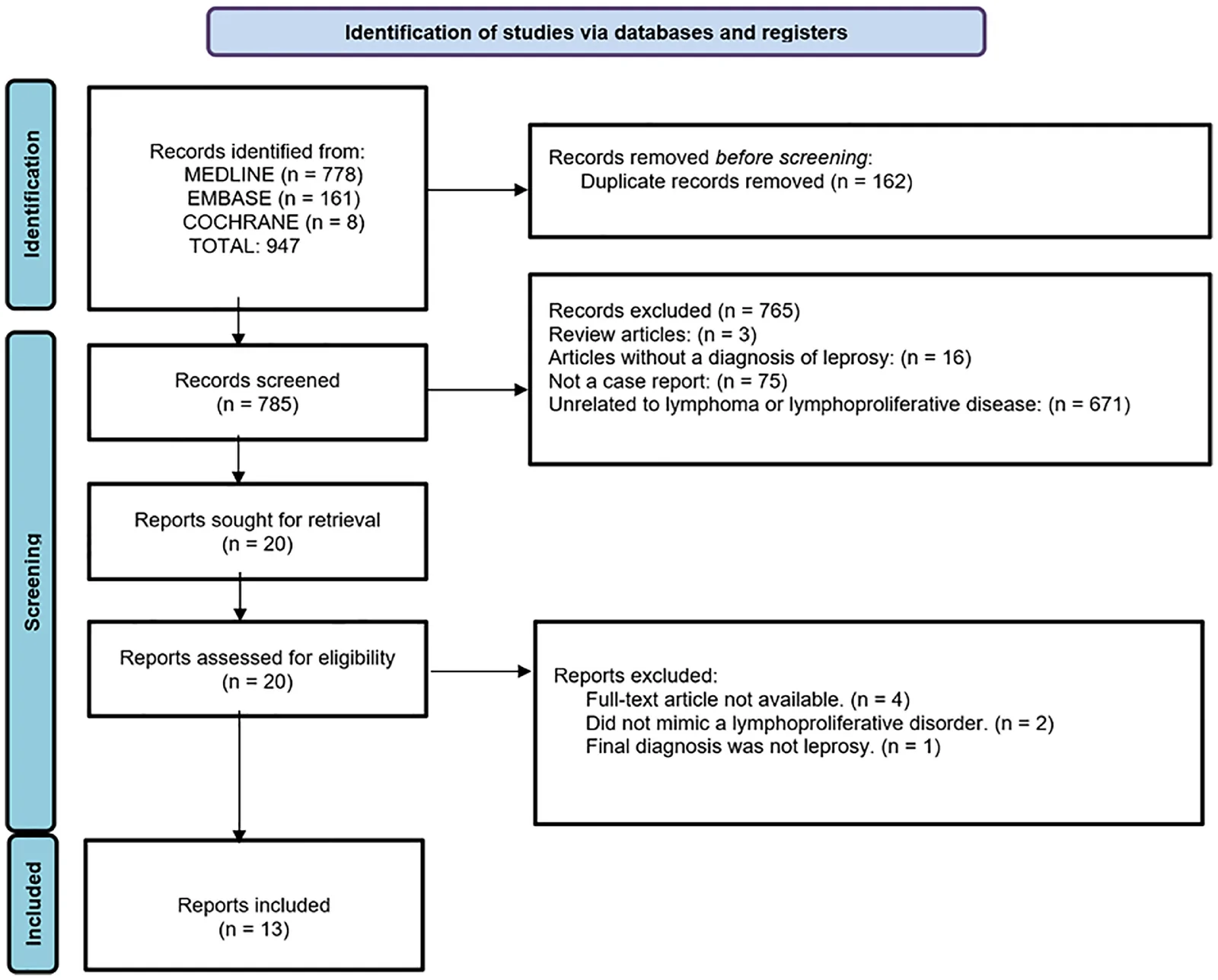
Flow diagram of the structured literature search and study selection process.

## Case presentation

A 44-year-old White woman from southern Brazil, employed in a shoe factory, was referred to the Internal Medicine outpatient clinic due to painful lymphadenopathy. The patient reported the onset of symptoms approximately 1 year prior (October 2023), starting with acute, intense stabbing pain (rated 9/10) in the right inguinal region. This was followed by the appearance of a palpable swelling, later identified as an enlarged lymph node. Over the following months, she experienced the progressive development of multiple lymphadenopathies involving the left inguinal chain and bilateral cervical and axillary chains. Initially painful, the lymph nodes became painless after a few days. Subsequently, she developed nonspecific widespread erythematous skin lesions, which began on the lower limbs and spread to the trunk, upper limbs, and face. These lesions were pruritic and occasionally associated with mild burning discomfort. In addition, she reported systemic symptoms including unintentional weight loss (approximately 9 kg over 6 months), night sweats, and daily episodes of fever, predominantly in the evening, with chills and malaise. One fever episode had been objectively measured (38.5°C in September 2024) (Figure 2 illustrates the timeline of symptom onset and progression).

**Figure 2:**
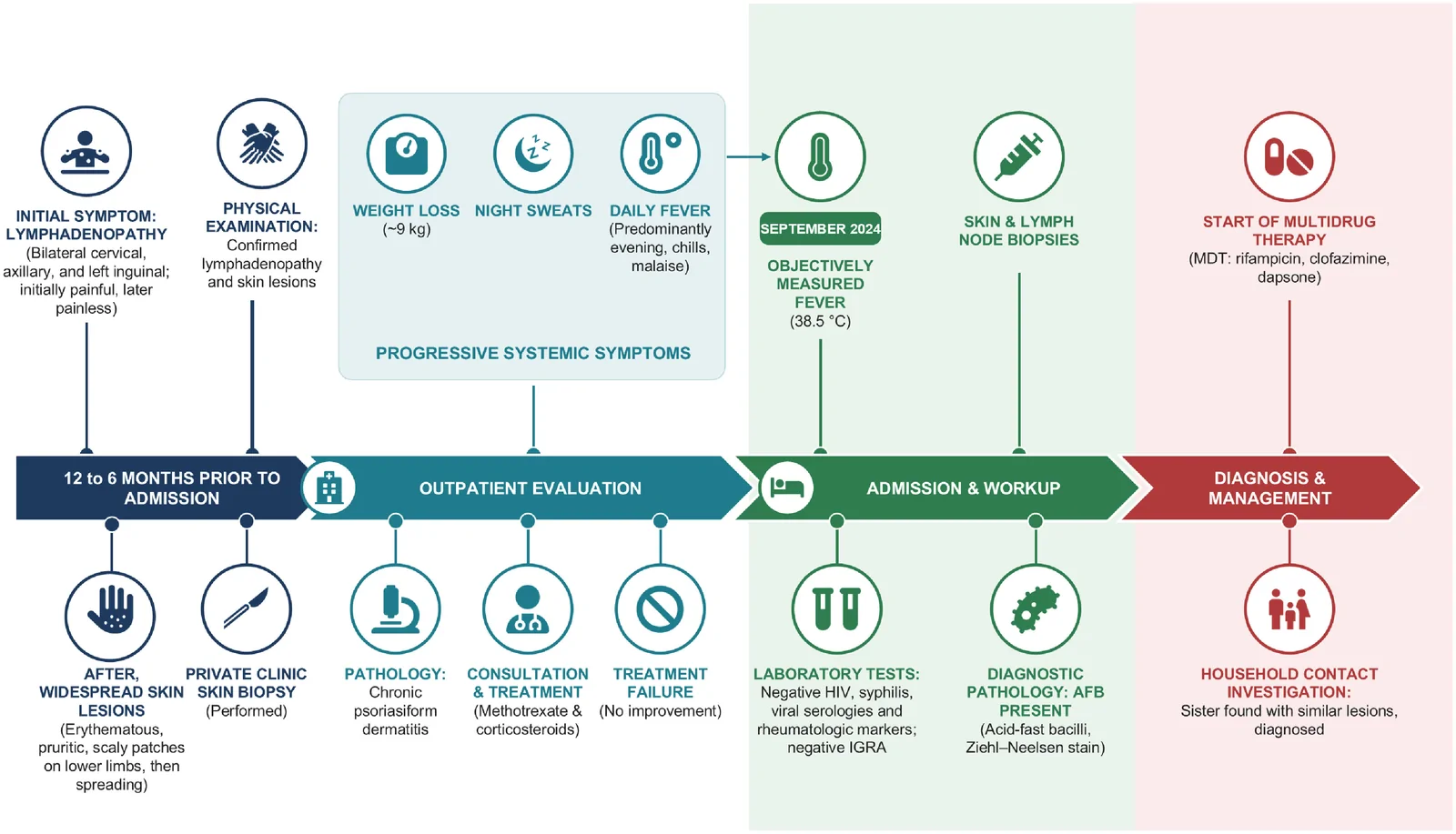
Clinical course and diagnostic journey of a patient with atypical Hansen's disease. Legend: Timeline illustrating the chronological progression of symptoms, diagnostic workup, and clinical management in a 44-year-old woman presenting with atypical leprosy. AFB, acid-fast bacilli; HIV, human immunodeficiency virus; IGRA, interferon-gamma release assay; MDT, multidrug therapy. The visual composition was generated with the assistance of ChatGPT, GPT-5.5 Thinking, using image-generation capabilities.

She had results from a skin biopsy previously performed on an outpatient basis at a private clinic, which showed “*chronic psoriasiform dermatitis with focal non-confluent parakeratosis and mild superficial perivascular inflammatory infiltrate.”* At the time, she consulted with a general practitioner in the countryside, who suspected psoriasis but, considering lymphadenopathy to be the most prominent clinical finding, referred the patient for further evaluation of a possible lymphoproliferative disorder. He prescribed methotrexate (2.5 mg six tablets per week) and corticosteroids, without improvement. At this point, the patient was referred to a specialized internal medicine service in southern Brazil for further investigation. She reported a smoking history (5 pack-years) and denied any known drug allergies or previous hospital admissions. The patient denied recent travel to regions endemic for infectious-contagious diseases and denied the presence of similar symptoms in other family members.

Physical examination at admission revealed a patient in good general condition. Vital signs were within normal ranges. Cardiopulmonary and abdominal exams were unremarkable. Multiple palpable lymph nodes were identified in the anterior and posterior cervical chains, bilateral axillary regions, and bilateral inguinal chains, measuring 1-2 cm in diameter, mobile, firm, and tender to palpation. Dermatological examination revealed erythematous, scaly skin lesions distributed across the lower limbs, trunk, upper limbs, and face, as illustrated in Figure 3a and b. Some lesions were excoriated due to scratching, and the patient reported pruritus and an occasional burning sensation.

**Figure 3:**
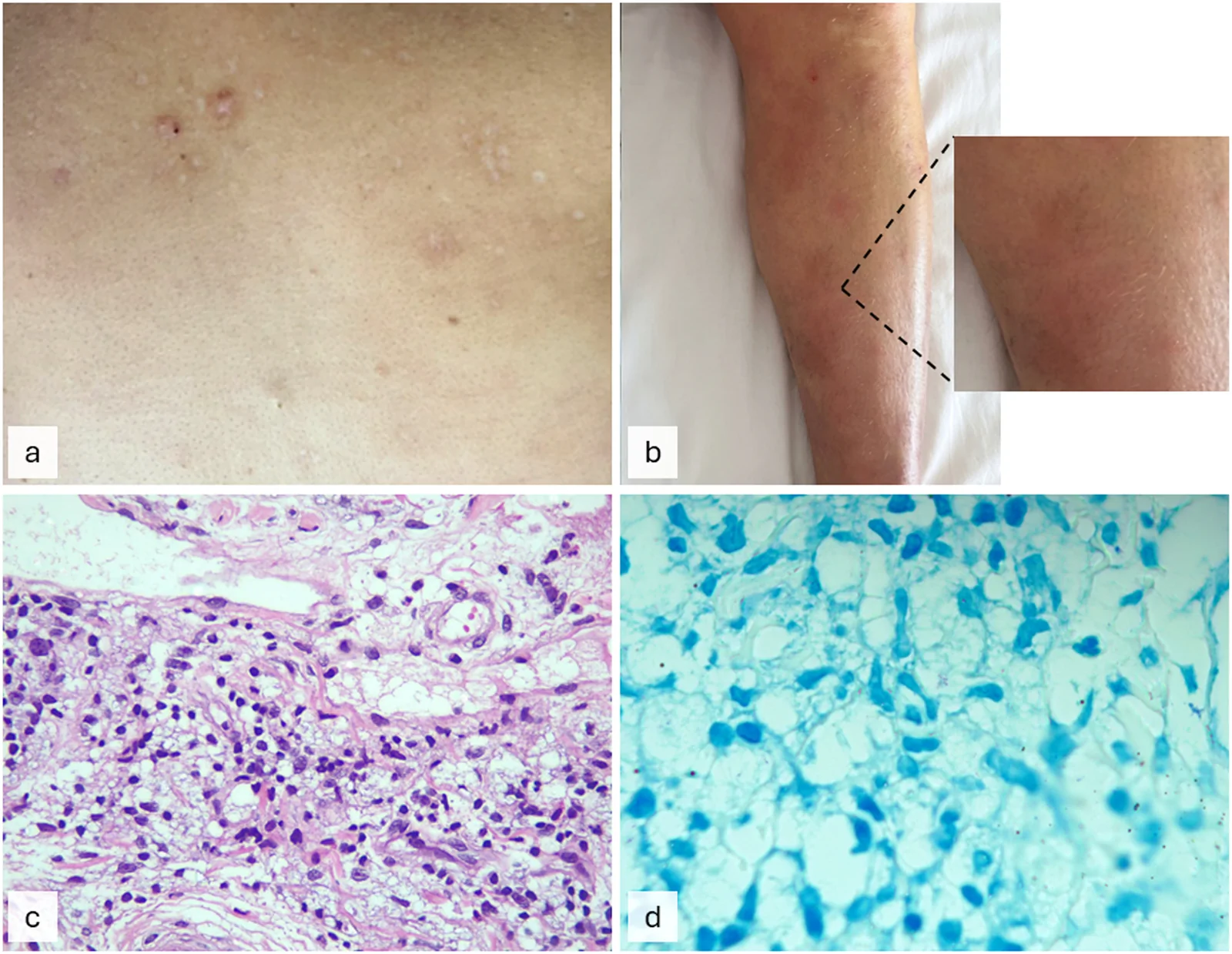
Dermatological and histopathological findings. Legend: **(a)** Dorsal view demonstrating multiple erythematous, hyperpigmented macules and papules scattered across the trunk, some with central clearing, consistent with multibacillary leprosy skin lesions. **(b)** Lower limb showing erythematous, subtly scaly lesions along the lateral aspect of the leg, representative of the widespread cutaneous involvement extending from the lower limbs to the trunk and upper extremities. Histopathological findings: **(c)** Hematoxylin and eosin (H&E) staining showing an infiltrate composed predominantly of foamy histiocytes (40 × objective; 400 × total magnification). **(d)** Ziehl–Neelsen staining demonstrating acid-fast bacilli within the histiocytic infiltrate (100 × oil-immersion objective; 1000 × total magnification). Photomicrographs obtained by pathologist Katiane Bortolini Zenatti.

Diagnostic workup was initiated. Laboratory tests (Supplementary Table 3) revealed mild anemia, and negative rheumatologic markers. Viral serological testing showed reactive cytomegalovirus (CMV) IgG with non-reactive CMV IgM, and reactive Epstein–Barr virus (EBV) anti-viral capsid antigen (VCA) IgG with non-reactive anti-VCA IgM, a profile consistent with past rather than acute infection. HIV serology and the Venereal Disease Research Laboratory (VDRL) test for syphilis were non-reactive. The interferon-gamma release assay for latent tuberculosis infection was negative. Imaging studies demonstrated axillary and retropectoral lymphadenopathy on chest computed tomography, as well as an increased number of cervical lymph nodes on neck computed tomography (Supplementary Table 4). Skin and lymph node biopsies were performed, with Ziehl–Neelsen staining demonstrating acid-fast bacilli. Histopathologic analysis of the skin biopsy revealed a “*perivascular and periannexal inflammatory infiltrate composed of histiocytes with clear, vacuolated cytoplasm, accompanied by lymphocytes, findings consistent with the clinical hypothesis of Hansen’s disease (leprosy),”* as illustrated in Figure 3c and d*.*

The patient was referred to the Sanitary Dermatology Clinic in Porto Alegre. Following diagnosis, a household contact investigation was conducted, during which her sister was found to have similar skin lesions clinically compatible with Hansen’s disease, prompting further evaluation and treatment.

The patient was classified as multibacillary and started uniform multidrug therapy (rifampicin 600 mg and clofazimine 300 mg supervised monthly; clofazimine 50 mg and dapsone 100 mg daily), completing 12 monthly doses.

The type 2 reaction (erythema nodosum leprosum) identified at diagnosis was accompanied by neuritis. Electroneuromyography, performed before the initiation of thalidomide, demonstrated sensorimotor axonal neuropathy of the lower limbs, with distal and predominantly sensory involvement; histopathological examination was compatible with leprosy-related neural involvement. Thalidomide was started at 400 mg/day and maintained for approximately 1 month until resolution of symptoms, then tapered by 50 mg every 2 weeks. Because of the associated neuritis, prednisone 1 mg/kg/day was administered concomitantly, with gradual reduction every 2 weeks and a total duration of 6 months, in accordance with national guidelines; acetylsalicylic acid 100 mg/day was prescribed as thromboembolic prophylaxis for the combination. Thalidomide was dispensed under the controlled framework established by Brazilian Health Regulatory Agency Resolution 11/2011. No type 1 (reversal) reaction occurred at any point during treatment or follow-up.

Skin lesions regressed progressively, with residual hyperchromic macules by month 6 of therapy. Constitutional symptoms (fever, arthralgia, weight loss, asthenia) resolved after starting treatment. Lymphadenopathy, considered part of the systemic spectrum of erythema nodosum leprosum, resolved partially: at last follow-up, a residual reactional node persisted in the inguinal chain. It is currently managed by clinical observation with serial examination.

Adherence was complete: all 12 supervised doses were administered on schedule, and self-administered doses were confirmed at each monthly visit. Treatment was well tolerated, with no dose reductions or interruptions. Neuropathic symptoms persisted after completion of uniform multidrug therapy and resolution of the reactional episode. Referral to neurology was requested for functional assessment and management of the residual neuropathy, including neuropathic pain control; at the time of submission, the patient is awaiting this appointment through the public health system. She remains under regular dermatology follow-up. Follow-up data reported here were obtained from outpatient dermatology records.

## Literature review and case discussion

This case illustrates the diagnostic challenge posed by Hansen's disease (leprosy) in a nonclassical presentation. The patient experienced a delay of approximately 1 year between symptom onset and definitive diagnosis, during which she was empirically treated for psoriasiform dermatitis without improvement. This diagnostic delay is not uncommon in leprosy, particularly in regions where the disease is not routinely considered in the differential diagnosis, and reflects the protean clinical manifestations of *M. leprae* infection. The constellation of progressive, painless lymphadenopathy across multiple chains, widespread erythematous skin lesions, and systemic B symptoms, including weight loss, night sweats, and fever, initially raised broad differential diagnoses, especially lymphoma.

Prior immunosuppressive therapy may have influenced the clinical expression and temporal evolution of disease in this patient. She had a history of prolonged and intermittent exposure to corticosteroids and other immunosuppressive agents, with repeated periods of treatment, discontinuation, and reintroduction before the diagnosis of leprosy. Corticosteroids exert clinically relevant immunomodulatory effects on leprosy reactions, including suppression of inflammatory mediators involved in type 1 reactions, and masking of leprosy manifestations during corticosteroid therapy has previously been reported [8,9]. Methotrexate has also been used as a corticosteroid-sparing immunomodulatory treatment for both type 1 reactions and erythema nodosum leprosum, with recurrence of leprosy reactions reported following its discontinuation [10]. These observations support the possibility that prolonged and fluctuating immunosuppression may have modified the clinical expression of leprosy in our patient. However, no clear temporal relationship between the withdrawal of immunosuppressive therapy and the onset or worsening of her manifestations could be established, and a causal relationship cannot be inferred.

Given the rarity of fever and lymphadenopathy as predominant initial manifestations of leprosy, and their potential to mimic lymphoma, we conducted a structured literature search to contextualize this patient’s presentation and diagnostic trajectory within previously published comparable cases. A total of 947 records were identified across MEDLINE, Embase, and Cochrane. Among the case reports identified through our structured literature search, 13 described leprosy presentations that were clinically or histopathologically mistaken for, or raised suspicion of, lymphoma or another lymphoproliferative disorder, reinforcing that this diagnostic pitfall, although rare, is a recurring theme across endemic and nonendemic settings alike [[11], [12], [13], [14], [15], [16], [17], [18], [19], [20], [21], [22], [23]]. Most of these reports share the same underlying mechanism as our patient: infiltration of lymph nodes and/or skin by *M. leprae*-laden macrophages produces painless, multi-chain lymphadenopathy and systemic constitutional symptoms (fever, night sweats, weight loss) that closely mimic the clinical picture of a lymphoproliferative malignancy, particularly in patients without the classical hypopigmented, anesthetic skin patches taught in textbook descriptions of leprosy.

The cases most similar to ours were those describing generalized, painless lymphadenopathy accompanied by a full constitutional (B-symptom-like) syndrome, in which leprosy was only reached after tuberculosis and/or lymphoma had already been considered or treated empirically. Mahajan et al. [12] described a 36-year-old man with 12 months of fever, generalized painful-then-painless lymphadenopathy, and skin lesions who was sequentially misdiagnosed with tuberculous lymphadenitis and Hodgkin's lymphoma before lepromatous leprosy with a type 2 reaction was confirmed. Herekar et al. [15] and Kiran et al. [16] reported comparable trajectories, in which chronic fever and lymphadenopathy were initially attributed to disseminated tuberculosis or tubercular lymphadenitis, with leprosy (and, in Kiran's case, a late bacteriological relapse) only identified after further biopsy. Bagla et al. [11] and Singh et al. [13] each described patients in whom lymph node enlargement generated a strong clinical suspicion of non-Hodgkin's lymphoma, with the diagnosis of lepromatous leprosy ultimately established by node histopathology or fine-needle aspiration cytology demonstrating lepra cells and bacilli. The closest parallel, however, is the recent Brazilian case reported by Reis et al. [14], which described inguinal lymphadenopathy with testicular pain, chills, insomnia, night sweats, and roughly 9 kg of weight loss over 2 months in a patient with multibacillary leprosy from the same endemic country, essentially replicating the systemic symptom profile and diagnostic delay seen in our patient.

A second cluster of reports resembled our case chiefly through its cutaneous rather than nodal presentation. Gunawan et al. [17] described a patient with reversal-reaction borderline lepromatous leprosy manifesting as psoriasis-like plaques over the trunk and limbs together with mycosis-fungoides-like tumors on the face, an important parallel given that our patient's outside skin biopsy had originally been read as "psoriasiform dermatitis" and treated accordingly with methotrexate without improvement. Nawalerspanya et al. [18] and Rodríguez et al. [19] similarly illustrated how histoid leprosy can produce nodular or prurigo-nodularis-like lesions that prompt a differential diagnosis of cutaneous lymphoma or other neoplasia, only resolved once Ziehl–Neelsen or Fite staining revealed abundant bacilli. These cases differ from ours mainly in lesion morphology (discrete nodules/tumors rather than diffuse pruritic erythematous plaques) and in the comparatively limited nodal involvement, but they underscore the same underlying message: unusual skin infiltrates in leprosy can be misclassified as lymphoproliferative or psoriasiform disease when histopathological suspicion for mycobacterial infection is low.

Taken together, our structured literature search identified relatively few published cases combining, as our patient did, disseminated multi-chain lymphadenopathy, a full constitutional syndrome, and an initial misdiagnosis. This combination underscores both the rarity of such a comprehensive presentation and the persistent risk of diagnostic delay when leprosy is not considered in patients from endemic regions presenting with lymphadenopathy and systemic symptoms, reinforcing the educational aim of this case report and structured literature search.

### Limitations and diagnostic challenges

This report has limitations inherent to its single-case design and does not allow causal inferences regarding the potential influence of prior immunosuppressive therapy on disease expression. In addition, the structured literature search is subject to publication and reporting bias and cannot establish the true frequency of this presentation. Follow-up data were obtained retrospectively from outpatient dermatology records, as direct contact with the patient could not be re-established after hospital discharge. During hospitalization, the patient expressed distress regarding the prolonged diagnostic process and the succession of ineffective treatments she had received before the diagnosis of leprosy. She also reported concern about the occupational impact of the facial skin lesions; however, her perspective on the post-treatment outcome could not be obtained. Characterization of the residual neuropathy also remains incomplete, as specialized neurological assessment was still pending at the time of submission, and the residual inguinal lymph node remains under clinical observation without a definitive outcome. Diagnostic delay in this case was likely multifactorial, reflecting the nonclassical cutaneous presentation, the initial histopathological interpretation as psoriasiform dermatitis, the absence of a recognized contact history, and the presence of generalized lymphadenopathy with constitutional symptoms that reasonably raised suspicion of a lymphoproliferative disorder. Earlier consideration of leprosy in the differential diagnosis, particularly in an endemic setting, together with targeted histopathological examination and acid-fast bacilli staining, might have shortened the diagnostic interval.

## Conclusion

This case highlights how leprosy can remain diagnostically elusive when it presents with atypical systemic and lymph node manifestations that closely resemble lymphoma. The prolonged diagnostic trajectory underscores a persistent gap in clinical awareness and training, particularly regarding nonclassical presentations of the disease. Failure to recognize leprosy early not only delays appropriate treatment and increases the risk of irreversible disability but also allows ongoing transmission, as illustrated by the identification of a household contact with skin lesions suggestive of the disease. These findings reinforce the need to strengthen education among health care professionals, especially in primary care and in endemic settings, while ensuring timely referral, systematic contact evaluation, and improved integration between clinical services and public health surveillance.

## Funding

This study was financed in part by the Coordenação de Aperfeiçoamento de Pessoal de Nível Superior-Brasil (CAPES) - Finance Code 001.

## Ethics approval and consent to participate

The patient provided signed informed consent for publication of this case report. All authors signed a confidentiality agreement regarding data use.

## Consent for publication

All authors have reviewed the final version of the manuscript and agree to the publication of the content presented.

## Declaration of generative AI and AI-assisted technologies in the writing process

During the preparation of this work, the authors LA and JA used ChatGPT (OpenAI) and Claude (Anthropic, Sonnet 4.6) as supportive tools for language editing and not as sources of scientific evidence. The visual composition of Figure 2 was generated with the assistance of ChatGPT using image-generation capabilities, based on an author-defined prompt designed to illustrate the patient’s clinical course and diagnostic journey. The authors reviewed and edited all artificial intelligence-assisted output as needed and take full responsibility for the content of the manuscript.

## CRediT authorship contribution statement

**Laís Agostinetto:** Writing – original draft, Methodology, Formal analysis, Data curation, Conceptualization. **Caroline Goergen:** Writing – original draft, Data curation. **Amanda Hillesheim Schuck:** Validation, Methodology, Formal analysis. **Gabriela de Oliveira Centofante:** Writing – review & editing, Writing – original draft, Data curation. **Katiane Bortolini Zenatti:** Methodology, Investigation. **Janine Alessi:** Writing – review & editing, Supervision, Investigation, Data curation, Conceptualization.

## Declaration of competing interest

The authors have no competing interests to declare. Author C.G. received a scholarship from CAPES. This study received no specific funding.

## Appendix Supplementary materials

Image, application 1
