# Supplementary material for: Leprosy mimicking lymphoproliferative disorders: a case report and structured literature search
Source: IJID Reg. 2026 Sep 26;21:101029. doi: 10.1016/j.ijregi.2026.101029 (PMC13694846; doi:10.1016/j.ijregi.2026.101029)
Supplement: Supplementary file 1 [file mmc1.docx]

**SUPPLEMENTARY MATERIAL**

Table 1. Laboratory tests

| Date | Exam | Results | Reference Range |
| --- | --- | --- | --- |
| 26/01/25 | Complete Blood Count | Red Blood Cell Count: 4.03 million/µL  Hemoglobin: 10.7 g/dL Hematocrit: 33.2%  Mean Corpuscular Volume: 82.4 fL  Mean Corpuscular Hemoglobin: 26.6 pg  Red Cell Distribution Width: 14.6%  Erythroblasts: 0%  White Blood Cell Count: 8,730/µL  Neutrophils: 6,801/µL  Basophils: 44/µL  Eosinophils: 140/µL  Monocytes: 637/µL  Lymphocytes: 1,109/µL  Platelet Count: 318,000/µL  Mean Platelet Volume: 9.4 fL | Red Blood Cell Count: 4.5–6.1 million/µL  Hemoglobin: 12.8–17.8 g/dL  Hematocrit: 39.0–53.0%  Mean Corpuscular Volume: 80.0–100.0 fL  Mean Corpuscular Hemoglobin: 27.0–32.0 pg  Red Cell Distribution Width: 11.5–14.5%  Erythroblasts: absent  White Blood Cell Count: 3,600–11,000/µL  Neutrophils: 1,500–7,900/µL  Basophils: 0–300/µL  Eosinophils: 0–800/µL  Monocytes: 100–1,100/µL  Lymphocytes: 1,000–5,500/µL  Platelet Count: 150,000–450,000/µL  Mean Platelet Volume: 8.1–12.5 fL |
| 26/01/25 | Urinalysis | PHYSICOCHEMICAL EXAM RESULT:  pH: 5.5  Specific Gravity: 1.012  Protein: Negative  Glucose: Negative  Ketone Bodies: Negative Hemoglobin: Positive (+)  Bilirubin: Negative Urobilinogen: Normal  Nitrite: Negative  SEDIMENT:  Epithelial Cells: 1/field (5.6/µL)  Leukocytes: <1/field (<5.5/µL)  Red Blood Cells: <5/field (27.8/µL)  Hyaline Casts: absent  Granular Casts: absent  Crystals: absent | PHYSICOCHEMICAL EXAM RESULT:  pH: 4.5–7.5  Specific Gravity: 1.005–1.030  Protein: Negative  Glucose: Negative  Ketone Bodies: Negative Hemoglobin: Negative  Bilirubin: Negative  Urobilinogen: Normal  Nitrite: Negative  SEDIMENT:  Epithelial Cells: <6/field (<31/µL)  Leukocytes: <5/field (<25/µL)  Red Blood Cells: <4/field (<23/µL)  Hyaline Casts: absent  Granular Casts: absent  Crystals: absent |
| 26/01/25 | C-Reactive Protein | 18.2 mg/dL | <1.0 mg/dL |
| 26/01/25 | Erythrocyte Sedimentation Rate | 113 mm/h | Men: <15 mm/h  Women: <20 mm/h |
| 26/01/25 | Serum Sodium | 137 mEq/L | 137–145 mEq/L |
| 26/01/25 | Serum Potassium | 3.7 mEq/L | 3.5–5.1 mEq/L |
| 26/01/25 | Ionized Calcium | 4.5 mg/dL | 4.6–5.4 mg/dL |
| 26/01/25 | Serum Lactate Dehydrogenase | 147 U/L | 120–246 U/L |
| 26/01/25 | Total Bilirubin | 0.3 mg/dL | 0–1.3 mg/dL |
| 26/01/25 | Direct Bilirubin | 0.2 mg/dL | 0–0.4 mg/dL |
| 26/01/25 | Indirect Bilirubin | 0.1 mg/dL | 0–0.9 mg/dL |
| 26/01/25 | Aspartate Aminotransferase | 25 U/L | 17–59 U/L |
| 26/01/25 | Alanine Aminotransferase | 26 U/L | 0–50 U/L |
| 26/01/25 | Serum Magnesium | 2.0 mg/dL | 1.6–2.3 mg/dL |
| 26/01/25 | Prothrombin Time (PT) | Time: 12.6 s  Activity: 88%  International Normalized Ratio (INR): 1.1 | Time: 10.2–13.5 s  Activity: >70%  International Normalized Ratio (INR): 0.9–1.2 |
| 26/01/25 | Activated Partial Thromboplastin Time (APTT) | Sample: 32.1 s  Control: 28 s | 23.5–32.6 s |
| 27/01/25 | C3 complement | 193 mg/dL | 90–170 mg/dL |
| 27/01/25 | C4 complement | 31 mg/dL | 12–36 mg/dL |
| 27/01/25 | P-ANCA | Non-reactive | Non-reactive |
| 27/01/25 | C-ANCA | Non-reactive | Non-reactive |
| 27/01/25 | Rheumatoid factor | 9 IU/mL | ≤14 IU/mL |
| 27/01/25 | Creatine Kinase (CK) | 33 U/L | 55–170 U/L |
| 27/01/25 | Aldolase | 3.1 U/L | ≤7.6 U/L |
| 27/01/25 | Serum Iron | 47 µg/dL | 37–110 µg/dL |
| 27/01/25 | Ferritin | 363 ng/mL | Men: 22–275 ng/mL  Women: 5–204 ng/mL |
| 27/01/25 | Total Iron-Binding Capacity (TIBC) | 295 µg/dL | 265–497 µg/dL |
| 27/01/25 | Transferrin Saturation | 16% | 20–50% |
| 27/01/25 | Vitamin B12 | 201.0 pg/mL | 239–931 pg/mL |
| 26/01/25 | HIV Serology | Non-reactive | Non-reactive |
| 26/01/25 | Venereal Disease Research Laboratory (VDRL) | Non-reactive | Non-reactive |
| 28/01/25 | Cytomegalovirus IgG Antibodies | Reactive | Non-reactive: <4.99 U/mL Indeterminate: 5.00–7.99 U/mL  Reactive: >8.00 U/mL |
| 28/01/25 | Cytomegalovirus IgM Antibodies | Non-reactive | Index >1.20: Reactive  Index 0.90–1.20: Indeterminate  Index <0.90: Non-reactive |
| 28/01/25 | Epstein–Barr Virus Anti-VCA IgM | Non-reactive | Index ≥1.00: Reactive  Index 0.75–0.99: Indeterminate Index <0.50: Non-reactive |
| 28/01/25 | Epstein–Barr Virus Anti-VCA IgG | Reactive | Index >1.10: Reactive  Index 0.90–1.10: Indeterminate Index <0.75: Non-reactive |
| 28/01/25 | Antinuclear Antibodies (ANA, HEp-2 cells) | Non-reactive | Non-reactive |
| 28/01/25 | Anti-DNA Antibody | Non-reactive | Non-reactive |
| 29/01/25 | Antistreptolysin O Antibody | 205 IU/mL | <140 IU/mL |
| 29/01/25 | Interferon-Gamma Release Assay (IGRA) | Non-reactive | Non-reactive |

Table 2. Imaging studies

| 26/01/25 | Neck CT | Neck CT revealed normal mucosal relief of the pharynx and larynx without evident lesion. Increased number of cervical lymph nodes, not meeting criteria for lymphadenopathy. Laryngeal cartilages intact. Trachea and esophagus without detectable abnormality. Parotid glands, submandibular glands, and thyroid gland with normal morphology and density. Cervical spaces preserved. Major cervical vessels patent. |
| --- | --- | --- |
| 26/01/25 | Chest CT | Small, scattered ground-glass opacities persist in the anterior segment of the right upper lobe and in the medial segment of the middle lobe, presumably post-inflammatory in nature. No evidence of consolidation or expansile lesion in the lungs. Trachea and main bronchi patent. No mediastinal or hilar lymphadenopathy identified. There was an increase in the number of axillary and retropectoral lymphadenopathies bilaterally, larger on the left side measuring up to 2.0 cm. Normal cardiac volume. Thoracic aorta of usual caliber, with parietal atheromatous plaques. Pulmonary artery trunk of usual caliber. No signs of coronary or aortic valve calcifications. No pleural or pericardial effusion. Evaluation of the bony structures demonstrates signs of degenerative disease. |
| 26/01/25 | Abdominal CT | There was an increase in the hypervascular nodular image in hepatic segment II, now measuring 1.5 cm (previously 1.1 cm), and a new lesion with similar characteristics appeared in segment V measuring 0.5 cm, both presumably benign in nature. Increase in hepatic dimensions. Gallbladder normally distended, without signs of radiopaque calculi. No intra- or extrahepatic biliary duct dilatation observed. Adrenal glands, pancreas, and spleen with usual tomographic appearance. Kidneys in normal position, enhancing with contrast and symmetrically excreting opacified urine. Cortical irregularities in the right kidney, presumably sequelae of a previous inflammatory process. Aorta with preserved caliber and discrete parietal calcifications. The common hepatic and splenic arteries originate directly from the aorta (anatomical variant). Small lymph nodes along the common iliac chain and retroperitoneum remain similar, measuring up to 1.3 × 0.7 cm. No signs of free fluid in the abdominal cavity. |
| 26/01/25 | Breast ultrasound | Fibroglandular and homogeneous background echotexture. Hypoechoic, oval, and circumscribed nodule measuring 0.6 cm in the upper outer quadrant of the left breast, presumably benign in nature. In the left axillary region, confluent lymphadenopathies are observed, the largest at level III with indistinct margins, measuring 2.8 cm. No other noteworthy findings were observed. Diagnostic impression: Suspicious left axillary lymphadenopathies. BI-RADS US 4. |
| 28/01/25 | Abdominal MRI | Hepatomegaly. Hepatic nodular lesions, presumably benign in nature (adenoma?). Area of probable perfusional alteration in hepatic segment VI. Sequelae cortical irregularities in the right kidney. |

Table 3. Systematic Review – search syntax

| **MEDLINE** | ( "Leprosy"[Mesh] OR leprosy[tiab] OR leprous[tiab] OR hansen*[tiab] OR "Hansen disease"[tiab] OR "Hansen's disease"[tiab] OR "Mycobacterium leprae"[tiab] OR "M. leprae"[tiab] OR "multibacillary leprosy"[tiab] OR "multibacillary Hansen disease"[tiab] OR "borderline leprosy"[tiab] OR "borderline lepromatous leprosy"[tiab] OR "borderline tuberculoid leprosy"[tiab] OR "borderline borderline leprosy"[tiab] OR "dimorphous leprosy"[tiab] OR "dimorphic leprosy"[tiab] OR "lepromatous leprosy"[tiab] ) AND ( "Lymphoma"[Mesh] OR "Lymphadenopathy"[Mesh] OR "Lymphatic Diseases"[Mesh] OR lymphoma*[tiab] OR "lymphoproliferative disorder"[tiab] OR "lymphoproliferative disorders"[tiab] OR lymphoproliferative[tiab] OR pseudolymphoma*[tiab] OR "pseudo-lymphoma"[tiab] OR "pseudo-lymphomas"[tiab] OR lymphadenopath*[tiab] OR "lymph node enlargement"[tiab] OR "enlarged lymph node"[tiab] OR "enlarged lymph nodes"[tiab] OR "lymph node involvement"[tiab] OR "lymph node lesion"[tiab] OR "lymph node lesions"[tiab] OR "lymph node biopsy"[tiab] OR "lymph node biopsies"[tiab] OR "hematologic malignancy"[tiab] OR "hematological malignancy"[tiab] OR "hematologic neoplasm"[tiab] OR "hematological neoplasm"[tiab] OR "malignant lymphoma"[tiab] OR "nodal disease"[tiab] OR "nodal involvement"[tiab] OR "systemic lymphadenopathy"[tiab] OR "generalized lymphadenopathy"[tiab] OR "cervical lymphadenopathy"[tiab] OR "mediastinal lymphadenopathy"[tiab] OR "retroperitoneal lymphadenopathy"[tiab] OR "mimicking lymphoma"[tiab] OR "mimicked lymphoma"[tiab] OR "masquerading as lymphoma"[tiab] OR "misdiagnosed as lymphoma"[tiab] OR "suspected lymphoma"[tiab] OR "lymphoma-like"[tiab] OR "lymphoma like"[tiab] ) AND ( "Case Reports"[Publication Type] OR "case report"[tiab] OR "case reports"[tiab] OR "case study"[tiab] OR "case studies"[tiab] OR "case series"[tiab] OR "case presentation"[tiab] OR "case presentations"[tiab] OR "a case of"[tiab] OR "we report"[tiab] OR "we describe"[tiab] OR "patient report"[tiab] ) NOT ( review[pt] OR systematic review[pt] OR meta-analysis[pt] ) |
| --- | --- |
| **EMBASE** | ('leprosy'/exp OR 'mycobacterium leprae'/exp OR leprosy:ti,ab,kw OR leprous:ti,ab,kw  OR hansen*:ti,ab,kw OR 'hansen disease':ti,ab,kw OR 'mycobacterium leprae':ti,ab,kw  OR 'm leprae':ti,ab,kw OR 'multibacillary leprosy':ti,ab,kw  OR 'multibacillary hansen disease':ti,ab,kw OR 'borderline leprosy':ti,ab,kw  OR 'borderline lepromatous leprosy':ti,ab,kw OR 'borderline tuberculoid leprosy':ti,ab,kw  OR 'dimorphous leprosy':ti,ab,kw OR 'dimorphic leprosy':ti,ab,kw  OR 'lepromatous leprosy':ti,ab,kw)  AND  ('lymphoma'/exp OR 'lymphadenopathy'/exp OR 'lymphatic system disease'/exp  OR lymphoma*:ti,ab,kw OR 'lymphoproliferative disorder':ti,ab,kw  OR 'lymphoproliferative disorders':ti,ab,kw OR lymphoproliferative:ti,ab,kw  OR pseudolymphoma*:ti,ab,kw OR 'pseudo-lymphoma':ti,ab,kw OR 'pseudo-lymphomas':ti,ab,kw  OR lymphadenopath*:ti,ab,kw OR 'lymph node enlargement':ti,ab,kw  OR 'enlarged lymph node':ti,ab,kw OR 'enlarged lymph nodes':ti,ab,kw  OR 'lymph node involvement':ti,ab,kw OR 'lymph node lesion':ti,ab,kw  OR 'lymph node lesions':ti,ab,kw OR 'lymph node biopsy':ti,ab,kw  OR 'lymph node biopsies':ti,ab,kw OR 'hematologic malignancy':ti,ab,kw  OR 'hematological malignancy':ti,ab,kw OR 'hematologic neoplasm':ti,ab,kw  OR 'hematological neoplasm':ti,ab,kw OR 'malignant lymphoma':ti,ab,kw  OR 'nodal disease':ti,ab,kw OR 'nodal involvement':ti,ab,kw  OR 'systemic lymphadenopathy':ti,ab,kw OR 'generalized lymphadenopathy':ti,ab,kw  OR 'cervical lymphadenopathy':ti,ab,kw OR 'mediastinal lymphadenopathy':ti,ab,kw  OR 'retroperitoneal lymphadenopathy':ti,ab,kw OR 'mimicking lymphoma':ti,ab,kw  OR 'mimicked lymphoma':ti,ab,kw OR 'masquerading as lymphoma':ti,ab,kw  OR 'misdiagnosed as lymphoma':ti,ab,kw OR 'suspected lymphoma':ti,ab,kw  OR 'lymphoma-like':ti,ab,kw OR 'lymphoma like':ti,ab,kw)  AND  ('case report'/exp OR 'case report':ti,ab,kw OR 'case reports':ti,ab,kw  OR 'case study':ti,ab,kw OR 'case studies':ti,ab,kw OR 'case series':ti,ab,kw  OR 'case presentation':ti,ab,kw OR 'case presentations':ti,ab,kw OR 'a case of':ti,ab,kw  OR 'we report':ti,ab,kw OR 'we describe':ti,ab,kw OR 'patient report':ti,ab,kw)  NOT  ('review'/it OR 'systematic review'/exp OR 'meta analysis'/exp) |
| **COCHRANE** | #1 MeSH descriptor: [Leprosy] explode all trees  #2 (leprosy OR leprous OR hansen* OR "Hansen disease" OR "Hansen's disease" OR "Mycobacterium leprae" OR "M leprae" OR "multibacillary leprosy" OR "multibacillary Hansen disease" OR "borderline leprosy" OR "borderline lepromatous leprosy" OR "borderline tuberculoid leprosy" OR "dimorphous leprosy" OR "dimorphic leprosy" OR "lepromatous leprosy"):ti,ab,kw  #3 #1 OR #2  #4 MeSH descriptor: [Lymphoma] explode all trees  #5 MeSH descriptor: [Lymphadenopathy] explode all trees  #6 (lymphoma* OR "lymphoproliferative disorder" OR "lymphoproliferative disorders" OR lymphoproliferative OR pseudolymphoma* OR "pseudo-lymphoma" OR lymphadenopath* OR "lymph node enlargement" OR "enlarged lymph node" OR "enlarged lymph nodes" OR "lymph node involvement" OR "lymph node lesion" OR "lymph node lesions" OR "lymph node biopsy" OR "lymph node biopsies" OR "hematologic malignancy" OR "hematological malignancy" OR "hematologic neoplasm" OR "hematological neoplasm" OR "malignant lymphoma" OR "nodal disease" OR "nodal involvement" OR "systemic lymphadenopathy" OR "generalized lymphadenopathy" OR "cervical lymphadenopathy" OR "mediastinal lymphadenopathy" OR "retroperitoneal lymphadenopathy" OR "mimicking lymphoma" OR "mimicked lymphoma" OR "masquerading as lymphoma" OR "misdiagnosed as lymphoma" OR "suspected lymphoma" OR "lymphoma-like" OR "lymphoma like"):ti,ab,kw  #7 #4 OR #5 OR #6  #8 #3 AND #7 |
